# Harnessing Arbuscular Mycorrhizal Symbiosis to Enhance Growth and Resilience to Combined Drought and Heat Stress in Lily (*Lilium* spp.)

**DOI:** 10.3390/plants15050767

**Published:** 2026-03-02

**Authors:** Hafiz Athar Hussain, Zhanhuai Liang, Shujaat Hussain, Jianghui Luo, Shunzhao Sui, Daofeng Liu

**Affiliations:** 1 Chongqing Engineering Research Center for Floriculture, Key Laboratory of Agricultural Biosafety and Green Production of Upper Yangtze River (Ministry of Education), College of Horticulture and Landscape Architecture, Southwest University, Chongqing 400715, China; atharhussain@swu.edu.cn (H.A.H.);; 2 Institute of Horticultural Sciences, University of Agriculture, Faisalabad 38040, Pakistan

**Keywords:** arbuscular mycorrhizal fungi (AMF), plant growth, antioxidant enzymes, ROS, drought, heat stress, *Lilium* spp.

## Abstract

Abiotic stresses such as drought and heat increasingly threaten plant growth and ornamental quality, particularly in climate-sensitive floricultural crops. Arbuscular mycorrhizal fungi (AMF) are known to enhance plant resilience under such conditions, yet their role in lilies remains insufficiently explored. In this study, we used a two-tier experimental approach to evaluate AMF-mediated benefits in lilies. First, different AMF strains, namely *Funneliformis mosseae* (FM), *Rhizophagus intraradices* (RI), *Rhizophagus irregularis* (RIG), *Claroideoglomus etunicatum* (CE), *Diversispora versiformis* (DV), and a mixed consortium (MIX), were screened for growth-promoting effects in two Lilium species, Taiwan lily and *Lilium* cv. Sorbonne, under non-stress conditions. Second, a selected AMF–host combination from the screening was evaluated to improve tolerance to drought, heat, and combined drought + heat stress. Among the tested strains, DV and MIX showed the most consistent improvements across key growth traits and root colonization. In the stress experiment, stress treatments reduced growth and physiological performance, particularly under combined drought + heat. AMF inoculation enhanced plant performance by improving shoot and root biomass, improving root system architecture, and leading to a higher chlorophyll content, greater relative water content, and enhanced flower traits. Biochemical analyses further revealed that AMF mitigated stress-induced oxidative damage by reducing reactive oxygen species (ROS) accumulation, as shown by reduced O_2_•^−^ and H_2_O_2_ staining. This reduction in oxidative stress was supported by increased activities of key antioxidant enzymes, indicating that AMF activate cellular defense mechanisms. These findings underscore the potential of AMF as a sustainable biotechnological tool for improving stress tolerance in lilies and enhancing floricultural productivity under climate-challenged environments.

## 1. Introduction

Lily (*Lilium* spp.) is one of the most widely cultivated ornamental bulbs and holds considerable commercial value as a cut flower and landscape plant [1,2]. However, its production has become increasingly vulnerable to the rapid rise in extreme climate events. Incidents of drought and heat are now occurring more frequently, and importantly, often overlap during the growing season [3]. This is particularly problematic because plants subjected to simultaneous drought and heat stress often fare worse than under individual stresses, and lilies (*Lilium* spp.), which typically develop limited root penetration and require consistent soil moisture to sustain their high transpiration and flower-forming demands, are especially vulnerable [4,5]. Under future climate projections, these combined stresses are expected to intensify, placing lily production at substantial risk [6].

Drought and heat stress each disrupt plant physiology in distinct ways, but together they create a much more complex and damaging environment [7]. Water deficit forces stomatal closure and restricts photosynthesis, while high temperatures accelerate respiration, destabilize membranes, and promote the extreme production of reactive oxygen species (ROS) [7,8]. When these stresses coincide, the resulting imbalance in carbon assimilation, oxidative pressure, and hormonal signaling is much greater than the sum of their individual effects [7,9]. In lilies, these impacts are reflected in reduced vegetative growth, hindered bulb development, and poor flower formation traits that directly determine market quality and grower profitability [10,11]. Traditional mitigation practices such as shading, mulching, or increased irrigation can offer short-term relief [12], but these approaches are not always feasible or sustainable, especially in regions facing chronic water scarcity.

Given these challenges, there is growing interest in biological strategies that can strengthen plant resilience from within. AMF, which form mutualistic associations with the roots of most terrestrial plant species, are particularly promising. Their extensive hyphal networks improve access to soil nutrients and water and can enhance root hydraulic conductivity [13,14]. Over the last decade, numerous studies have shown that AMF can help plants cope with drought and heat by regulating osmotic balance, boosting antioxidant enzyme activities, and influencing phytohormonal responses, particularly those involving abscisic acid (ABA) and jasmonates [15,16,17,18]. AMF colonization has also been linked to the improved maintenance of photosynthetic pigments and reduced lipid peroxidation when plants are exposed to harsh environmental conditions [16,19].

While AMF have been widely studied in major crops such as maize, wheat, and tomato, their role in ornamental bulb crops has not been explored thoroughly. Despite these advances, the role of AMF in mitigating combined drought and heat stress in lilies remains underexplored. While AMF inoculation improves drought tolerance in *Cinnamomum migao*, which belongs to the laurel family, no studies have evaluated the efficacy of *Lauraceae* [20] under the simultaneous high-temperature and moisture limitation conditions increasingly common in open-field production. Moreover, cultivar-specific responses and optimal inoculation protocols, e.g., fungal species, timing, and dosage, are unknown, limiting practical adoption. Given lilies’ economic importance and physiological vulnerability, harnessing AMF symbiosis offers a promising, eco-friendly strategy to sustain production amid climate uncertainty.

To address the increasing vulnerability of lilies to concurrent drought and heat stress, this study employed a two-tiered experimental approach. In experiment 1, we screened multiple AMF strains for their growth-promoting potential in two commercially important lily cultivars, ‘Sorbonne’ and ‘Taiwan’, under normal conditions. Then experiment 2 focused on a selected AMF–host combination, based on Experiment 1, in enhancing stress resilience subjected to combined drought and heat stress. We hypothesized that mycorrhizal symbiosis would (i) improve plant growth and root vitality, (ii) enhance photosynthetic efficiency and water retention, and (iii) stimulate antioxidant and osmolyte-based defenses under drought and heat stress conditions. The findings are expected to provide physiological and biochemical insights for integrating AMF into climate-resilient ornamental horticulture.

## 2. Materials and Methods

### 2.1. Experiment 1: Screening of Arbuscular Mycorrhizal Fungi (AMF)

#### 2.1.1. AMF Inoculum Preparation

Five arbuscular mycorrhizal (AM) fungi strains were used in this study: *Rhizophagus intraradices* (R.I.), *Funneliformis mosseae* (F.M.), *Diversispora versiformis* (D.V.), *Claroideoglomus etunicatum* (C.E.), and *Rhizophagus irregularis* (R.Ig). The inocula of *R. intraradices*, *F. mosseae*, and *D. versiformis* were obtained from the Institute of Root Biology, Yangtze University, China, while *C. etunicatum* and *R. irregularis* were provided by the Institute of Plant Nutrition and Resource Environment, Beijing Academy of Agriculture and Forestry Sciences, Beijing, China.

The AMF were propagated using Maize (*Zea mays* L.) as the host plant in a substrate of local soil and river sand (1:1, *v*/*v*). The soil was collected from the Xiema Experimental Base, College of Horticulture and Landscape Architecture, Southwest University, Chongqing, China, and sieved through a 1 cm mesh to remove large stones and debris. The substrate was sterilized by dry heat at 160 °C for 4 h to eliminate indigenous microorganisms and spores, then cooled and Mixed at a 1:1 (*v*/*v*) ratio. After 90 days of maize cultivation, the aboveground parts were removed, and the root systems and substrate were harvested, homogenized, and used as inoculum. Spores were extracted from the substrate via wet sieving and 50% sucrose centrifugation [21] and quantified under a stereomicroscope (SMZ1000; Nikon, Tokyo, Japan). The mean spore densities per gram of substrate were as follows: *R. intraradices* 15 spores g^−1^, *F. mosseae* 12 spores g^−1^, *D. versiformis* 19 spores g^−1^, *C. etunicatum* 11 spores g^−1^, and *R. irregularis* 20 spores g^−1^. Seven inoculation treatments were established: (1) no mycorrhiza (NM), (2) *Funneliformis mosseae* (FM), (3) *Rhizophagus intraradices* (RI), (4) *Rhizophagus irregularis* (RIG), (5) *Claroideoglomus etunicatum* (CE), (6) *Diversispora versiformis* (DV), and (7) Mixed inoculum (MIX; equal spore ratio of all five strains).

#### 2.1.2. Plant Material and Experimental Setup

In this study, two lilies (*Lilium*) were used: the Taiwan lily (*Lilium formosanum*) and *Lilium* ‘Sorbonne’. The *L. formosanum* bulbs were obtained from Xiajiashan Baihui Flower and Tree Nursery, Puning, China, and the *L.* ‘*Sorbonne*’ bulbs were obtained from Chenggong Qiaoqiao Florist, Kunming, China.

Uniform bulbs were transplanted into plastic pots (14.5 × 17.5 × 12.2 cm) filled with a sterilized growth medium composed of peat, vermiculite, and perlite in equal proportions (1:1:1, *v*/*v*). The substrate was sterilized by autoclaving at 121 °C and 0.11 MPa for 1 h, followed by oven-drying at 120 °C for 2 h. The physicochemical characteristics of the medium were as follows: total nitrogen, 4.50 g kg^−1^; total phosphorus, 0.50 g kg^−1^; total potassium, 6.56 g kg^−1^; available nitrogen, 229.19 mg kg^−1^; available phosphorus, 173.97 mg kg^−1^; available potassium, 815.88 mg kg^−1^; organic matter, 339.85 g kg^−1^; and pH 5.7.

Prior to transplantation, roots were gently removed from the original substrate, rinsed with distilled water, surface-sterilized in 75% ethanol for 12 s, and rinsed again. Each pot received one plant and 1 L of substrate. For AMF treatments, 600 spores were inoculated per pot, distributed evenly around the root zone before covering with substrate.

All pots were maintained in a greenhouse at the Floriculture Laboratory, Southwest University, Chongqing, China, with 28 °C/22 °C (day/night) and 65–75% relative humidity. Standard horticultural management was practiced throughout the experimental period. Each treatment was replicated three times, with two plants per replicate (one plant per pot), for a total of six plants per treatment.

#### 2.1.3. Determination of Plant Morphological Indicators

Morphological characteristics were assessed at the flowering stage. Plant height, root length, number of leaves, number of flower buds, bulblets, and flower bulbs were manually recorded. Additional parameters such as flower diameter, bud length and width, bulb perimeter, crown width, and petiole diameter were measured using a measuring tape and digital caliper (T304B. W-1215, Shanghai Total Industrial Co., Ltd., Shanghai, China). For biomass determination, shoot and root tissues were oven-dried at 70 °C for 72 h and weighed to obtain the shoot dry weight (SDW) and root dry weight (RDW). Mycorrhizal colonization was assessed by clearing root segments (~1 cm in length) in 10% KOH at 90 °C for 60 min, followed by acidification in 1% HCl, and staining with 0.05% Trypan Blue in lactoglycerol [22]. Root samples were mounted on slides and examined under a light microscope (EX20; SOPTOP, Ningbo, China) at 400× magnification. At least 30 segments per replicate were scored using the gridline intersection method following the Giovannetti and Mosse method [23]. The total mycorrhizal colonization percentage, as well as the arbuscule and vesicle percentages, were calculated based on the number of colonized intersections relative to the total observed.

### 2.2. Experiment 2: Effects of AMF on Drought and Heat Stress

#### 2.2.1. Experimental Design

Based on the results of exp 1, Taiwan lily (*Lilium formosanum*) and the Mixed AMF inoculum (Mix) were selected for further evaluation under stress conditions. This selection was based on overall performance across key growth and reproductive traits and root colonization. The AMF inoculum was the same Mixed substrate used in exp 1, consisting of maize root fragments, spores, and hyphae. The substrate preparation and sterilization procedures followed those described in Experiment 1. This experiment was conducted in the Floriculture Laboratory at Southwest University, Chongqing, China.

#### 2.2.2. Stress Treatments

Before transplantation, seedling roots were surface sterilized with 75% ethanol for 10 s, thoroughly rinsed with distilled water, and transplanted into seedling pots that had been pre-treated with potassium permanganate solution. AMF inoculation was performed, applying 600 spores per pot evenly around the root zone. Non-inoculated plants served as the control (−M).

A total of four stress treatments were established in Experiment 2: (1) control (Ck, no stress), (2) drought (D), (3) heat (H), (4) combined heat + drought (HD), with mycorrhiza (+M) and without mycorrhiza (−M) inoculation. Each treatment was replicated three times, with two plants per replicate (one plant per pot), for a total of six plants per treatment, and all plants were grown in a growth chamber (RDN-1000D-4; Ningbo Dongnan Instrument Co., Ltd., Ningbo, China). Initially, pots were maintained at 25/18 °C (day/night) under a 14/10 h light/dark photoperiod and 60% relative humidity, with the light intensity set to 14,000 lx using the chamber control system. Standard horticultural practices were applied uniformly across all treatments.

At the budding stage, plants subjected to heat and combined stress were shifted to high-temperature conditions of 38 °C/28 °C with a 12/12 h light/dark photoperiod, while maintaining the same light and humidity. Drought stress was imposed by maintaining soil moisture at 50% of field capacity (FC) on the basis of the gravimetric/pot-weight method. For the combined HD treatment, both temperature and drought stresses were applied simultaneously. The control (Ck) and drought (D) treatments continued under the initial temperature conditions. Twenty days after the onset of the stress treatments, plant growth data were recorded, and plant samples were collected for physiological parameters.

#### 2.2.3. Determination of Plant Growth Indicators and Root Activity

Morphological traits were recorded following the same procedure as in Experiment 1. Root activity refers to root respiratory/dehydrogenase activity, measured using the 2,3,5-triphenyltetrazolium chloride (TTC) reduction assay as described by Clemensson-Lindell [24] with slight modifications. Fresh root tips (0.5 g) were incubated in 0.4% TTC prepared in phosphate buffer (pH 7.0) at 37 °C in darkness for 1–3 h. The reaction was terminated by adding 2 mL of 1 M H_2_SO_4_, and the red formazan (TTF) produced was extracted with TCA. Absorbance was measured at 485 nm using Varioskan Flash; Thermo Scientific, Vantaa, Finland. Root activity, expressed as TTC-reducing intensity (µg g^−1^ h^−1^), was calculated as:

Root activity = C/m × t

where C is the amount of TTC reduced (µg), m is the fresh root mass (g), and t is the incubation time (h).

#### 2.2.4. Determination of Mycorrhizal Colonization

To assess mycorrhizal colonization, root samples were stained with trypan blue using the method described by Phillips and Hayman [22]. The extent of colonization was then quantified using the “Mycocalc” software (https://www2.dijon.inra.fr/mychintec/Mycocalc-prg/download.html (accessed on 16 May 2025)), based on the approach developed by Trouvelot et al. [25]. This analysis provided values for the frequency of mycorrhizal infection (F%), the overall intensity of colonization (M%), and the abundance of arbuscules (A%) within the root system. Additionally, the total percentage of mycorrhizal colonization was calculated.

#### 2.2.5. Determination of Physiological Parameters

Photosynthetic pigments were extracted and quantified following a modified protocol described by Wu [26]. Fresh leaf samples (0.1 g) were extracted in 10 mL of acetone–ethanol (1:1, *v*/*v*) and kept in darkness at 25 °C for 24 h. After centrifugation, 200 µL of the clear extract was used to measure absorbance at 663, 645, and 470 nm using a microplate reader (Varioskan Flash; Thermo Scientific, Vantaa, Finland). The concentrations of chlorophyll a, chlorophyll b, and total chlorophyll were determined using the following equations:

Chla = 12.72A_663_ − 2.59A_645_

Chlb = 22.88A_645_ − 4.67A_663_

Chl total = 8.02A_663_ + 20.21A_645_

Pigment content was then normalized to tissue fresh weight using the formula:

Pigment content (mg·g^−1^ FW) = C × V/(W × 1000),

where C is the pigment concentration (mg L^−1^), V is the extraction volume (mL), and W is the fresh tissue weight (g).

Relative water content (RWC) was determined following Barrs and Weatherley [27]. Fresh leaf discs (~0.5 g) were weighed (FW), hydrated in distilled water for 4 h to record turgid weight (TW), and oven-dried at 70 °C for 48 h to obtain dry weight (DW). RWC was calculated as:

RWC (%) = (FW − DW)/(TW − DW)× 100

Lipid peroxidation was assessed by measuring the malondialdehyde (MDA) content following the method of Campos et al. [28] with slight modifications. Briefly, leaf tissue (0.5 g) was homogenized in 10 mL of 5% (*w*/*v*) trichloroacetic acid (TCA) and centrifuged (Sorvall ST8R; Thermo Fisher Instruments Co., Ltd., Suzhou, China) at 4000 rpm for 10 min. Equal volumes (2 mL each) of supernatant and 5% TCA containing 0.67% thiobarbituric acid (TBA) were mixed, heated at 100 °C for 15 min, cooled rapidly, and centrifuged again. Absorbance was measured at 450, 532, and 600 nm, and the MDA concentration was computed as:

MDA (μmol/L) = 6.45(A_532_ – A_600_) − 0.56A_450_.

The MDA content per gram of fresh tissue was expressed as:

MDA (nmol g^−1^ FW) = C × V/(1000 × W).

where C is the MDA concentration (μmol/L), V is the volume of extract (mL), and W is the sample fresh weight (g).

Electrolyte leakage (EL), representing cell membrane stability, was determined by incubating 0.5 g of leaf tissue in 30 mL of deionized water at room temperature for 3 h with gentle shaking (100 rpm). The initial conductivity (S_1_) was recorded using a conductivity meter (DDS-307W; Shanghai Yueping Scientific Instrument Co., Ltd., Shanghai, China). The samples were then boiled for 30 min to release total electrolytes, cooled, and the final conductivity (S_2_) was measured. Relative electrolyte leakage was calculated as described by Campos et al. [28] using the formula:

EL (%) = (S1 × 100)/S2.

#### 2.2.6. Reactive Oxygen Species (ROS) Quantification and Histochemical Staining

Superoxide anion (O_2_•^−^) and hydrogen peroxide (H_2_O_2_), two key reactive oxygen species (ROS), were evaluated using a combination of histochemical staining and spectrophotometric (Varioskan Flash; Thermo Scientific, Vantaa, Finland) quantification. Fully expanded leaves were excised and vacuum-infiltrated with 10 mM of potassium phosphate buffer (pH 7.8) for 30 min. O_2_•^−^ accumulation was visualized using nitroblue tetrazolium (NBT) staining following the method of Grellet Bournonville and Díaz-Ricci [29] with minor modifications. Leaf tissues were incubated in 1 mg/mL NBT solution (10 mM phosphate buffer, pH 7.8) at 25 °C in darkness for 12 h until blue formazan formation. O_2_•^−^ content was measured following Wang and Luo [30] with minor modifications. Approximately 0.2 g of fresh leaf tissue was homogenized in 5 mL of 65 mM phosphate buffer (pH 7.8), centrifuged (Sorvall ST8R; Thermo Fisher Instruments Co., Ltd., Suzhou, China) at 8000× *g* for 20 min, and reacted with hydroxylamine hydrochloride (10 mM). The mixture was further incubated with 17 mM of sulfanilic acid and 7 mM of α-naphthylamine, and absorbance was recorded at 530 nm after pigment removal using anhydrous ether.

H_2_O_2_ accumulation and content were detected according to Guan et al. [31], with slight modification. Leaves were immersed in 1 mg/mL DAB (3,3′-diaminobenzidine) solution (10 mM potassium phosphate buffer, pH 7.8) and incubated at 25 °C in darkness for 5 h. Following staining, the samples were decolorized by boiling in bleaching solution (ethanol: acetic acid: glycerol Mixture, 3:1:1, *v*/*v*/*v*) until all Chl was completely removed. Stained leaves were photographed using a digital camera (EOS Rebel SL1; Canon Inc., Tokyo, Japan).

For H_2_O_2_ content, 0.5 g of leaf tissue was homogenized in 5 mL of ice-cold acetone, centrifuged (Sorvall ST8R; Thermo Fisher Instruments Co., Ltd., Suzhou, China) at 10,000 *g* for 10 min, and the supernatant was analyzed spectrophotometrically (Varioskan Flash; Thermo Scientific, Vantaa, Finland). H_2_O_2_ concentration was determined using a standard curve generated with 0.1–100 nM analytical-grade H_2_O_2_ (30%).

#### 2.2.7. Detection of Antioxidants and Osmolytes

Superoxide dismutase (SOD) activity was assayed according to Dhindsa et al. (1981) with minor adjustments [32]. About 0.2 g of fresh leaf tissue was homogenized in 0.1 M phosphate buffer (pH 7.5) containing 0.5 mM of EDTA, followed by centrifugation at 10,000 rpm for 15 min at 4 °C. The 3 mL reaction mixture included 13 mM of methionine, 75 μM of NBT, 0.1 mM of EDTA, 1.3 μM of riboflavin, phosphate buffer, and 0.1 mL of enzyme extract. After 15 min of illumination, absorbance was measured at 560 nm. One enzyme unit corresponded to 50% inhibition of NBT reduction.

Peroxidase (POD) activity was assessed using guaiacol and H_2_O_2_ as substrates according to Putter [33]. Approximately 0.5 g of leaf tissue was extracted in 5 mL of 50 mM phosphate buffer (pH 7.8), centrifuged at 15,000 rpm for 20 min at 4 °C, and the supernatant was used for assays. The 3 mL reaction mixture (pH 7.0) consisted of 10 mM of guaiacol, 5 mM of H_2_O_2_, and 0.2 mL of enzyme extract. Absorbance was read at 470 nm at 1 min intervals for 5 min, and one unit represented a 0.01 increase in A_470_ per minute.

Catalase (CAT) activity was determined following Aebi [34]. The 3 mL reaction contained 100 mM of H_2_O_2_ and 50 mM of phosphate buffer (pH 7.8). After adding 0.2 mL of enzyme extract, the decline in A_240_ was recorded every minute for 4 min. One unit of CAT corresponded to a 0.01 decrease in absorbance per minute.

The free proline content was quantified using the acid ninhydrin method [35]. Fresh tissue (0.5 g) was extracted in 3% sulfosalicylic acid, reacted with ninhydrin and acetic acid, and heated at 100 °C for 30 min. The toluene phase was separated and measured at 520 nm against a L-proline standard curve.

Soluble sugar content was measured by the anthrone-sulfuric acid method [36]. Leaf tissue (0.2 g) was homogenized in distilled water, centrifuged, and reacted with anthrone reagent. Absorbance was read at 620 nm and sugar concentration was calculated using a glucose standard.

Total soluble protein was estimated by the Bradford method [37]. About 0.25 g of leaf tissue was homogenized in phosphate buffer, centrifuged, and the supernatant was mixed with Bradford reagent. Absorbance was determined at 595 nm.

#### 2.2.8. Statistical Analysis

Each experiment followed a completely randomized design (CRD) with three replications (n = 3). Data analysis was performed using Statistix 8.1 software. Data were analyzed using two-way ANOVA to test the main effects of AMF inoculation and stress treatment, as well as their interaction (AMF*Stress). Mean comparisons were conducted using Tukey’s test at *p* < 0.05. Data visualization and graphing were performed using Origin 2024.

## 3. Results

### 3.1. Effect of Different Mycorrhizal Strains on the Growth of Lilium Species

AMF inoculation was associated with improved shoot growth in both *Lilium* genotypes relative to the non-inoculated control (Table S1). In Taiwan lily, all AMF treatments led to a notable increase in plant height, averaging a 12% improvement over the control (CK). Among the treatments, DV and MIX strains exhibited the strongest effects, increasing plant height by 16% and 18%, respectively. Although stem diameter remained statistically unchanged across treatments, shoot dry weight showed a moderate improvement in most AMF-inoculated plants, with FM and MIX performing better than the control, with 27% and 20% improvements, respectively. Notably, the number of leaves increased under the influence of AMF, with DV 42% and MIX 62% outperforming all others, highlighting a pronounced enhancement in foliar development.

In *Lilium* cv. Sorbonne, AMF application also resulted in increases in plant height, shoot biomass, and stem diameter (Table S1). The tallest plants were recorded in the DV (27%) and RIG (17%) treatments, compared with the control treatment. Meanwhile, shoot dry weight peaked in the RIG 56% and DV 50% treatments compared with the control treatments. Stem diameter was highest in DV (18%) and MIX (14%). Unlike the Taiwan lily, the number of leaves was less responsive to AMF, with no significant changes across treatments, although slight increases were seen in FM, RI, and MIX.

### 3.2. Effect of Different Mycorrhizal Strains on Root and Bulb Traits of Lilium Species

AMF inoculation affected multiple root and bulb traits in both genotypes (Figure S1; Table S2).

In Taiwan lily, root length was increased under FM 47%, MIX 40%, and RIG 37%, as compared to the control (CK), respectively. Root dry weight also improved with RI 24%, DV 30%, RIG 27%, and MIX 28%, all showing higher biomass accumulation than CK, although FM showed only marginal improvement, 4%. Notably, bulb perimeter expanded most in the MIX treatment, 36%, as compared with CK, indicating enhanced storage organ development. The number of bulblets, an important reproductive and economic trait, was highest in MIX 260% and DV 220%, suggesting that diverse or functionally synergistic AMF communities more effectively promote bulblet formation in the Taiwan lily.

In *Lilium* cv. Sorbonne, AMF also positively influenced root and bulb traits, though with different magnitudes (Table S2). Root length was nearly doubled in MIX 92% and CE 84% compared to CK, highlighting a strong AMF effect on root elongation. Root dry weight increased most in FM 45% and CE 43%, as compared with the control. Bulb perimeter remained relatively stable across all treatments, showing that AMF had less pronounced effects on bulb girth in Sorbonne than in Taiwan lily. However, the bulblet number significantly increased under DV 178%, MIX 136%, and FM 127%, as compared with CK, reinforcing the reproductive benefits of mycorrhizal symbiosis.

### 3.3. Effects of Different Mycorrhizal Strains on Floral Traits of Lilium Species

AMF inoculation was associated with enhanced floral traits in both genotypes (Table 1). In Taiwan lily, the number of flower buds increased 132% in the MIX treatment, as compared with the control. Flower bud length was most increased by RI 13%, DV 16%, and MIX 22%, as compared with the control. Similarly, bud width increased markedly under DV 42% and MIX 34% compared to CK. Flower diameter expanded with AMF, particularly under MIX 41% and DV 32%, indicating improved floral quality, as compared with the control. Overall, DV and MIX produced the most positive effects on flower size and bud production.

Sorbonne plants showed even stronger floral responses to AMF inoculation (Table 1). The highest number of buds was observed under DV 60% and MIX 54%, compared with CK. Bud length increased notably under MIX 33% and RIG 31%, while bud width was most increased by the MIX treatment 53%, as compared with the control. Flower diameter improved with AMF, reaching its maximum under MIX 37% and RIG 29%, as compared to CK.

Overall, *Lilium* ‘Sorbonne’ produced larger buds and flowers than the Taiwan lily, reflecting inherent varietal characteristics. AMF inoculation substantially boosted floral traits in both species, with the MIX treatment consistently producing superior outcomes, followed by DV and RIG. These findings suggest strong synergistic effects among AMF consortia and highlight their potential for enhancing ornamental quality, flower size, and reproductive performance in lily cultivation.

### 3.4. Effects of Mycorrhizal Strains on Root Colonization of Lilium Species

Mycorrhizal colonization varied across AMF treatments (Table 2). In Taiwan lily, MIX inoculum resulted in the highest colonization, with 73% arbuscules, 82% vesicles, and 89% total colonization, indicating strong synergistic colonization potential when multiple AMF strains are combined. DV also induced high colonization with 50% arbuscules, 68% vesicles, and 75% total, followed by FM and RI. In contrast, CE exhibited significantly lower colonization metrics with arbuscules (29%), vesicles (47%), and total (51%), suggesting a weaker symbiotic establishment in the Taiwan lily.

Similarly, the MIX inoculum yielded the highest total colonization (79%) and vesicle abundance 63% in ‘Sorbonne’, although arbuscule development (39%) was slightly lower than in Taiwan lily. FM, RI, and DV showed moderate colonization levels, while CE consistently showed the lowest values across all parameters: arbuscules (20%), vesicles (36%), and total (42%), highlighting its relatively poor colonization efficacy in both genotypes (Table 2). These findings confirm the superior symbiotic performance of multi-strain AMF inocula in both *Lilium* species and provide a strong foundation for selecting optimal fungal partners for improved growth.

Overall, MIX produced the strongest responses in Taiwan lily, combining the highest root colonization with marked increases in leaf and bulblet growth relative to the control. In contrast, the Sorbonne was comparatively less responsive across AMF treatments, so the Taiwan lily was used for the subsequent stress experiment to provide clearer treatment differentiation.

### 3.5. Summary of Treatment Effects Based on Two-Way ANOVA

To provide an overall statistical summary of the screening experiment, a two-way ANOVA was conducted to evaluate the effects of AMF treatment, lily genotype, and their interaction across the measured traits (Table S3). Overall, the lily genotype showed significant effects on most growth and reproductive parameters. AMF treatment significantly influenced shoot dry weight, leaf number, root length, bulblet and flower bud traits, and root colonization indices. Significant AMF × genotype interactions were detected for shoot dry weight, leaf number, root length, and colonization traits, viz., arbuscules and vesicles, indicating genotype-dependent responsiveness to AMF inoculation.

### 3.6. Effects of Arbuscular Mycorrhizal Fungi (AMF) on Morphological Traits of Taiwan Lily Under Drought and Heat Stress

**Figure 1:**
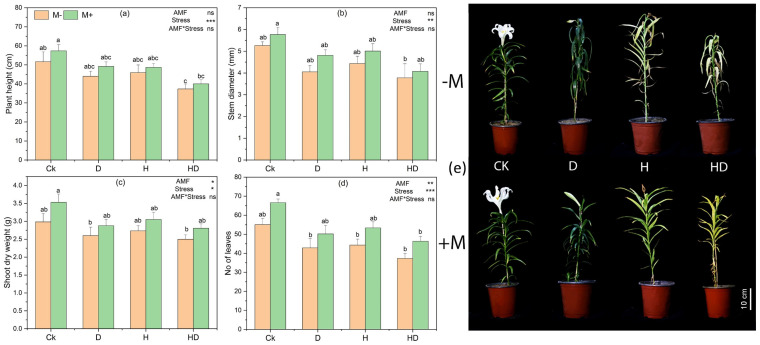
Effects of arbuscular mycorrhizal fungi (AMF) on aboveground morphological traits of Taiwan lily under control (Ck; no stress), drought (D), heat (H), and combined drought + heat (HD) stress with (+M) and without (−M) AMF inoculation. (**a**) Plant height (cm), (**b**) Stem diameter (mm), (**c**) Shoot dry weight (g), (**d**) Number of leaves, (**e**) Representative images of lily plants under each treatment. Bars represent mean ± standard error (n = 3). Different lowercase letters above bars indicate significant differences among treatments at *p* < 0.05 based on Tukey’s HSD test. The statistical significance of AMF, stress, and their interaction is shown in the upper right corner of each panel (* *p* < 0.05, ** *p* < 0.01, *** *p* < 0.001; ns, not significant).

Mycorrhizal inoculation generally improved plant performance under both control and stress conditions (Figure 1), whereas drought (D), heat (H), and combined drought + heat (HD) reduced aboveground growth relative to the control (CK), with the strongest reduction observed under HD (Figure 1e). However, the most significant reduction in plant height occurred between CK − M and HD − M with 27% (Figure 1a). For stem diameter, CK + M was significantly higher than HD − M with 29% (Figure 1b). For shoot dry weight, CK + M was significantly higher than D − M and HD − M with 21% and 24%, respectively (Figure 1c). For leaf number, CK + M was significantly higher than all stress treatments, with an average of 17% (Figure 1d). Although AMF-inoculated plants often showed higher mean values than non-inoculated plants within the same stress treatment, several of these differences were not statistically significant according to Tukey’s test (Figure 1).

### 3.7. Effects of Arbuscular Mycorrhizal Fungi (AMF) on Root Traits of Taiwan Lily Under Drought and Heat Stress

**Figure 2:**
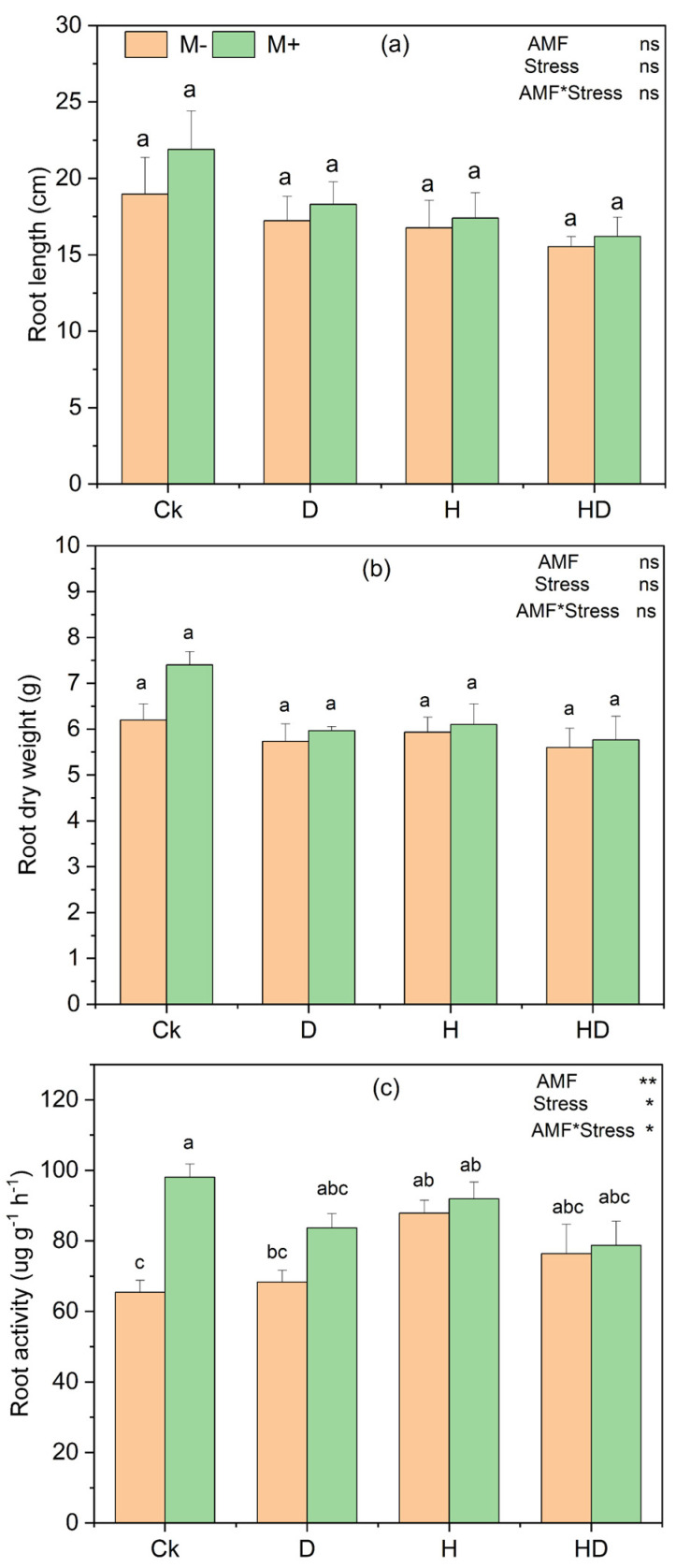
Effects of arbuscular mycorrhizal fungi (AMF) on root traits of Taiwan lily under control (Ck; no stress), drought (D), heat (H), and combined drought + heat (HD) stress with AMF (M+) and without AMF (M−) inoculation. (**a**) Root length (cm), (**b**) Root dry weight (g), and (**c**) Root activity (µg g^−1^ h^−1^). Different lowercase letters above bars indicate significant differences among treatments at *p* < 0.05 using Tukey’s HSD test. The statistical significance of main effects and their interaction is indicated in the top right of each panel (* *p* < 0.05, ** *p* < 0.01; ns, not significant).

The application of AMF showed modest effects on the root length and dry weight of Taiwan lily under drought, heat, and combined stress conditions. Although AMF-inoculated plants (M+) had a slightly higher root length and biomass than non-inoculated (M−), the differences were not statistically significant (Figure 2a,b). This suggests that root elongation and dry matter accumulation were relatively stable under stress and were not strongly modulated by AMF.

However, root respiratory/dehydrogenase activity, as measured by TTC-based root activity, showed a significant treatment response (Figure 2c). Pairwise comparisons indicated that AMF significantly increased root activity under control conditions (Ck + M vs. Ck − M), whereas under drought (D), heat (H), and combined drought + heat (HD), AMF-inoculated plants showed higher mean root activity than non-inoculated plants, but these differences were not statistically significant (Tukey’s HSD). Overall, these data indicate that AMF primarily enhanced root activity under non-stress conditions, with non-significant positive trends under stress.

### 3.8. Arbuscular Mycorrhizal Symbiosis with Taiwan Lily Under Drought and Heat Stress

**Figure 3:**
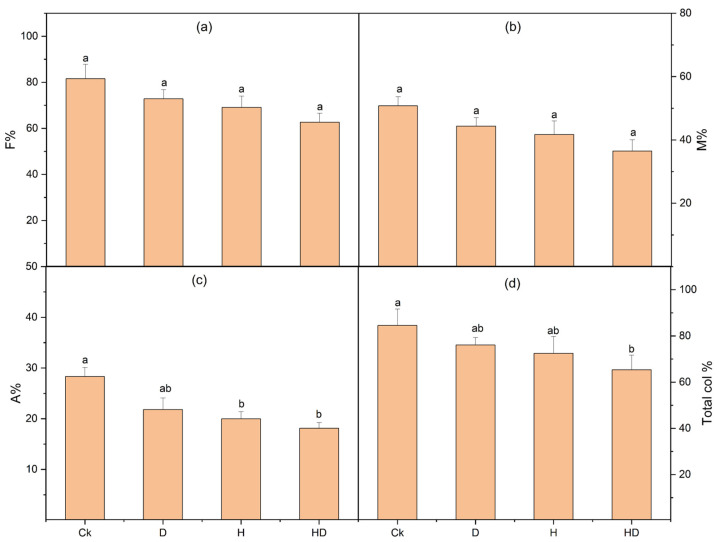
Effect of control (Ck; no stress), drought (D), heat (H), and combined drought + heat (HD) stress on AMF colonization parameters in Taiwan lily plants inoculated with arbuscular mycorrhizal fungi (AMF). (**a**) Frequency of colonization (F%), (**b**) Mycorrhizal intensity (M%), (**c**) Arbuscule abundance (A%), and (**d**) Total colonization percentage. Bars represent the mean ± standard error (n = 3). Different lowercase letters indicate statistically significant differences at *p* < 0.05 based on Tukey’s HSD test.

The extent of mycorrhizal colonization in Taiwan lily roots was significantly influenced by environmental stress conditions (Figure 3). Under control (Ck) treatment, plants exhibited the highest frequency (F%) and intensity (M%) of AMF colonization, exceeding 80% and 60%, respectively. Although drought (D) and heat (H) stress did not significantly reduce F% and M% values individually, a modest decline was observed compared to the control. Under combined drought + heat stress (HD), both F% and M% dropped more noticeably, suggesting additive inhibitory effects of concurrent stresses on mycorrhizal symbiosis.

Arbuscule abundance (A%) followed a similar trend, with the highest levels in control plants (28%), significantly higher than those in heat and combined stress treatments, which dropped to ~20% and ~18%, respectively. These reductions suggest compromised functional arbuscule development under thermal and hydric stress. The total colonization percentage showed a clear decreasing pattern with increasing stress severity. The combined stress treatment (HD) led to a significant reduction in colonization compared to the control, while drought and heat individually had a moderate impact. These findings suggest that while Taiwan lily maintains considerable AMF colonization under individual stress, simultaneous drought and heat stress significantly compromise colonization efficiency and arbuscular development, potentially affecting the symbiotic benefits conferred by AMF under extreme environmental conditions.

### 3.9. Effects of Arbuscular Mycorrhizal Fungi (AMF) on Chlorophyll and Relative Water Content of Taiwan Lily Under Drought and Heat Stress

**Figure 4:**
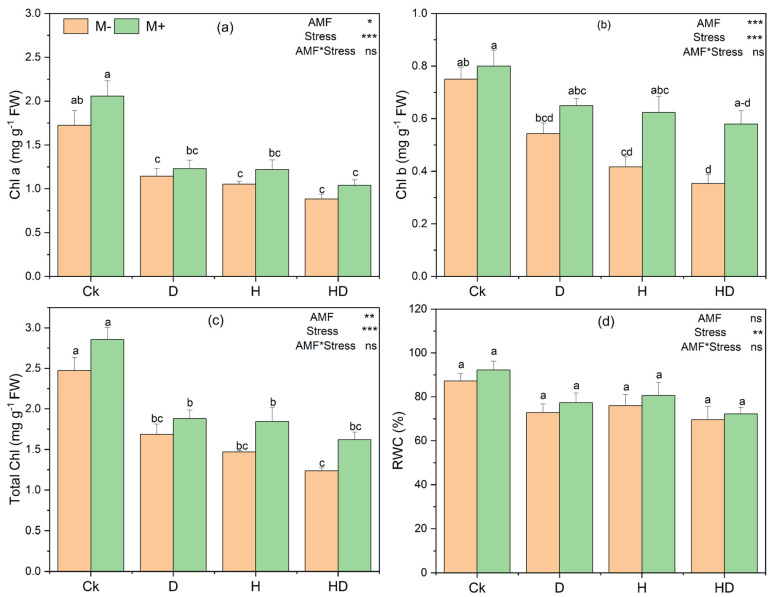
Effect of arbuscular mycorrhizal fungi (AMF) on leaf chlorophyll content and relative water content (RWC) in Taiwan lily under control (Ck; no stress), drought (D), heat (H), and combined drought + heat (HD) stress with AMF (M+) and without AMF (M−) inoculation. (**a**) Chlorophyll a (Chl a), (**b**) Chlorophyll b (Chl b), (**c**) Total chlorophyll (Chl), (**d**) Relative water content (RWC). Bars represent the mean ± standard error (n = 3). Different lowercase letters denote significant differences among treatments at *p* < 0.05 using Tukey’s HSD test. Statistical significance of main effects and their interaction is indicated in the top right of each panel (* *p* < 0.05, ** *p* < 0.01, *** *p* < 0.001; ns, not significant).

The physiological responses of Taiwan lily plants, particularly chlorophyll content and relative water content (RWC), were markedly affected by drought and heat stress, as well as AMF inoculation (Figure 4). Chlorophyll a (Chl a) and Chlorophyll b (Chl b) levels were reduced under all stress treatments compared with the control (Ck), with the lowest values generally observed under HD (Figure 4a–c). Two-way ANOVA indicated significant main effects of stress on pigment contents and significant main effects of AMF on Chl a, Chl b, and total chlorophyll (Figure 4a–c). However, Tukey’s HSD pairwise comparisons did not detect significant differences between M+ and M− within the same stress treatment, as most corresponding bars shared letter groupings. Thus, AMF-inoculated plants tended to show higher mean pigment levels than non-inoculated plants, but these increases were not consistently statistically supported at the within-treatment level.

RWC showed a significant main effect of stress (Figure 4d), while AMF and the AMF and stress interaction were not significant. Consistent with this, Tukey’s test indicated no significant differences among treatments for RWC (Figure 4d), although minor non-significant trends were observed.

### 3.10. Effects of Arbuscular Mycorrhizal Fungi (AMF) on Lipid Peroxidation and Membrane Damage of Taiwan Lily Under Drought and Heat Stress

**Figure 5:**
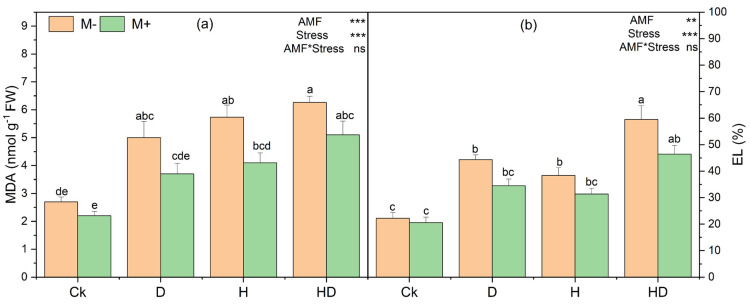
Effects of arbuscular mycorrhizal fungi (AMF) on (**a**) malondialdehyde (MDA) content and (**b**) electrolyte leakage (EL) in Taiwan lily under control (Ck; no stress), drought (D), heat (H), and combined drought + heat (HD) conditions, with (M+) and without (M−) AMF inoculation. Bars represent mean ± standard error (n = 3). Different lowercase letters above bars indicate significant differences at *p* < 0.05 according to Tukey’s HSD test. Statistical significance of main effects and their interaction is indicated in the top right of each panel (** *p* < 0.01, *** *p* < 0.001; ns, not significant).

Under drought (D), heat (H), and combined drought + heat (HD), both the malondialdehyde (MDA) content and electrolyte leakage (EL) increased relative to the control (Ck), indicating enhanced lipid peroxidation and membrane damage (Figure 5). The highest values were generally observed under HD, particularly in non-inoculated plants. Two-way ANOVA indicated significant main effects of stress and AMF on both MDA and EL (Figure 5a,b), while the AMF × stress interaction was not significant, suggesting that AMF effects were broadly consistent across treatments. However, Tukey’s HSD pairwise comparisons did not show consistent significant differences between M+ and M− within the same stress treatment, as the corresponding bars frequently shared letter groupings. Thus, AMF-inoculated plants tended to show lower mean MDA and EL values than non-inoculated plants under stress, but these differences were not reliably supported at the within-treatment level by Tukey’s test.

### 3.11. Effects of Arbuscular Mycorrhizal Fungi (AMF) on Reactive Oxygen Species (ROS) Accumulation in Taiwan Lily Under Drought and Heat Stress

**Figure 6:**
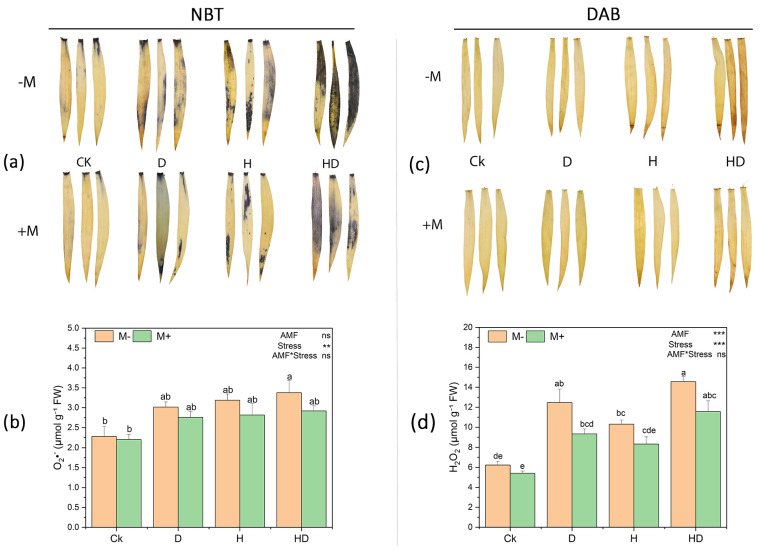
Effects of arbuscular mycorrhizal fungi (AMF) on reactive oxygen species (ROS) accumulation in Taiwan lily under control (Ck; no stress), drought (D), heat (H), and combined drought + heat (HD) conditions, with (M+) and without (M−) AMF inoculation. (**a**) Histochemical detection of ROS in leaves using nitro blue tetrazolium (NBT) for superoxide radicals (O_2_•^−^) and 3,3′-diaminobenzidine (DAB) for hydrogen peroxide (H_2_O_2_), (**b**) Quantification of O_2_•^−^ (**c**) Histochemical detection of hydrogen peroxide (H_2_O_2_) using 3,3′-diaminobenzidine (DAB) staining, (**d**) Quantification of H_2_O_2_. Bars represent mean ± standard error (n = 3). Different lowercase letters above bars indicate significant differences at *p* < 0.05 according to Tukey’s HSD test. Statistical significance of main effects and their interaction is indicated in the top right of each panel (** *p* < 0.01, *** *p* < 0.001; ns, not significant).

To evaluate oxidative stress responses, histochemical staining and the biochemical quantification of reactive oxygen species (ROS) were performed (Figure 6). Superoxide radicals (O_2_•^−^) were visualized by NBT staining, which showed stronger blue precipitates under stress treatments, particularly under combined drought + heat (HD), compared with the control (Figure 6a). Consistent with this, quantitative analysis indicated that O_2_•^−^ levels increased under drought (D), heat (H), and HD relative to CK (Figure 6b). Two-way ANOVA showed a significant effect of stress (*p* < 0.01), whereas the main effect of AMF and the AMF × stress interaction were not significant (Figure 6b). Accordingly, although M+ plants often showed lower mean O_2_•^−^ values than M− plants within the same stress treatment, these differences were not statistically significant based on Tukey’s HSD letter groupings.

Hydrogen peroxide (H_2_O_2_) accumulation, assessed by DAB staining, also intensified under stress, with more pronounced brown staining under HD compared with Ck (Figure 6c). Quantitative measurements confirmed that H_2_O_2_ content increased significantly under stress treatments (Figure 6d). Two-way ANOVA indicated significant main effects of both AMF and stress (*p* < 0.001), while the interaction was not significant (Figure 6d). However, within individual stress treatments, M+ and M− groups frequently shared Tukey letter groupings, indicating that AMF-associated reductions in H_2_O_2_ within the same stress condition were not consistently significant at the pairwise level. Overall, Figure 6 shows that drought and heat stress increased ROS accumulation, while AMF inoculation was associated with lower mean ROS levels in several treatments.

### 3.12. Effects of Arbuscular Mycorrhizal Fungi (AMF) on Antioxidant and Osmoprotectant Responses in Taiwan Lily Under Drought and Heat Stress

**Figure 7:**
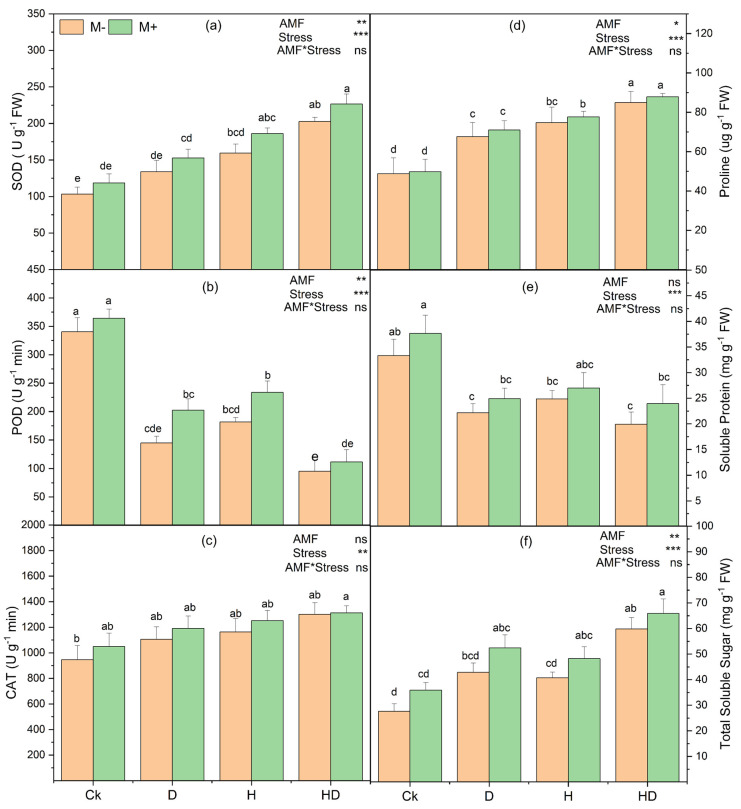
Effect of arbuscular mycorrhizal fungi (AMF) on antioxidant and osmoprotectant responses in Taiwan lily under control (Ck; no stress), drought (D), heat (H), and combined drought + heat (HD) conditions, with (M+) and without (M−) AMF inoculation. (**a**) Superoxide dismutase (SOD) activity, (**b**) Peroxidase (POD) activity, (**c**) Catalase (CAT) activity, (**d**) Proline content, (**e**) Soluble protein content, (**f**) Total soluble sugar content. Different lowercase letters above bars indicate significant differences at *p* < 0.05 according to Tukey’s HSD test. Statistical significance of main effects and their interaction is indicated in the top right of each panel (* *p* < 0.05, ** *p* < 0.01, *** *p* < 0.001; ns, not significant).

Drought (D), heat (H), and combined drought–heat (HD) stresses triggered significant physiological responses in Taiwan lily, particularly in antioxidant activity and osmolyte accumulation (Figure 7). AMF colonization played a critical role in modulating these responses.

SOD activity increased from Ck to HD, with the highest values observed under HD (Figure 7a). Mycorrhizal plants (M+) showed higher SOD activity compared to non-mycorrhizal controls (M−), indicating enhanced superoxide radical detoxification. POD activity showed an opposite trend, with the highest activity under Ck and reduced activity under stress treatments, reaching the lowest values under HD (Figure 7b). CAT activity also differed across treatments, with higher values under stress relative to Ck (Figure 7c). Across these enzyme assays, M+ plants often showed higher mean values than M− plants within the same stress condition; however, in most cases, the corresponding bars shared Tukey letter groupings, indicating that M+ vs. M− differences within a given stress treatment were not consistently significant.

Proline content increased under stress, with HD showing the highest levels relative to Ck (Figure 7d). The soluble protein content also varied among treatments, with the highest values observed under Ck and reduced levels under D, H, and HD (Figure 7e). Total soluble sugars increased under stress, with the highest values under HD (Figure 7f). Two-way ANOVA indicated significant effects of stress on all measured parameters, while AMF effects were significant for some traits, but the AMF × stress interaction was not significant (Figure 7). Taken together, Figure 7 demonstrates great stress-driven changes in antioxidant and osmoprotectant responses, with AMF-associated increases observed in several treatments, although pairwise M+ vs. M− differences were not consistently supported by Tukey’s test.

## 4. Discussion

Drought and heat are among the most damaging environmental stresses restricting plant growth and productivity, especially when they occur simultaneously [8,38]. Despite their importance, the biochemical and physiological responses of lilies to these stresses, and how they may be improved through beneficial root symbionts, have received limited attention. The AMF application helps plants improve their growth and overcome the stress effects [19,20]. This study clearly demonstrates the positive effect of AMF on the growth and development of *Lilium* species (Table 1, Table 2 and Tables S1–S3). AMF colonization improved plant performance, with notable differences observed between the Taiwan lily and *Lilium* cv. Sorbonne. These differences may reflect the inherent genetic variability in host symbiont compatibility and stress response.

In exp 1, AMF inoculation improved plant height, shoot biomass, root length, and the number of bulblets in both species. *Diversispora versiformis* (DV) and the mixed AMF consortium (MIX) were particularly effective (Table 1, Table 2 and Tables S1–S3), supporting previous findings that AMF-mediated growth promotion is strain-dependent [39,40]. Based on the screening results, we advanced Taiwan lily to the stress experiment because it showed the most consistent AMF responsiveness and strongest root colonization. MIX inoculum was selected for the stress phase, as it provided the most consistent overall improvements in growth and reproductive traits, making it the most suitable inoculum for stress validation.

Mycorrhizal inoculation influences the plant’s morphological, physiological, and biochemical processes, playing a crucial role in enhancing the growth and overall productivity of the host plant [41]. Several studies have previously demonstrated the negative effects of combined drought and heat stress on the growth and production of various plants [42,43]. However, the level of damage under these stresses varies depending on the intensity of stress and plant growth stage [44,45]. Under stress conditions, AMF improved both shoot and root growth performance, promoting shoot biomass and leaf growth, and enhancing root morphology and physiological activity (Figure 1, Figure 2 and Figure S2). Mycorrhizal colonization patterns revealed strong strain- and genotype-specific differences, with mixed AMF consortia achieving superior colonization (Table 2). In contrast, combined drought and heat stress reduced colonization intensity and arbuscule formation, potentially limiting symbiotic efficiency under extreme conditions (Figure 3).

The AMF develop arbuscules and extensive hyphal networks within roots and the surrounding soil, greatly expanding the effective root surface area and thereby enhancing nutrient uptake and overall plant growth [46]. In this study, plants treated with AMF showed increased chlorophyll levels and a higher relative water content (RWC), reflecting improved photosynthetic performance and water retention capacity (Figure 4). These traits are essential for plants to survive drought and heat stress [38]. Similarly, Zhang et al. (2018) also observed that inoculation with mycorrhiza enhanced *Lolium perenne* performance by increasing chlorophyll levels, photosynthetic efficiency, and fluorescence-related traits [47]. It was noted that drought decreased the photosynthesis, PSII activity, and chlorophyll pigment synthesis; however, AMF inoculation effectively mitigated these reductions, maintaining better photosynthetic function under stress [48]. The enhanced RWC observed in AMF-inoculated plants also supports the role of AMF in maintaining plant hydraulic conductivity and osmotic balance during water deficit conditions [49]. High temperatures accelerate chlorophyll degradation, which reduces light absorption and helps limit excessive free radical formation, but ultimately leads to impaired photosynthesis and plant damage [50]. Heat stress and drought may cause excessive ROS production, which damages plants’ photosynthetic components [38]. An indicator for lipid peroxidation is MDA concentration, which increases in plants because of the overproduction of ROS under unfavorable environmental conditions [50].

In this study, MDA, electrolyte leakage, and ROS were increased under heat and drought treatments, and AMF application decreased these accumulations (Figure 5 and Figure 6). However, the reduced accumulation of O_2_^−^ and H_2_O_2_, as revealed by NBT and DAB staining, highlights the role of AMF in mitigating oxidative stress (Figure 6). This is further supported by elevated activities of antioxidant enzymes and osmolytes in mycorrhizal plants (Figure 7). SOD scavenges O_2_^−^ whereas H_2_O_2_ is detoxified by CAT in the cytosol and by POD in the membranes [51]. These enzymes are part of the plant’s defensive system against ROS-induced cell damage [52]. Our findings are consistent with earlier reports indicating that AMF enhance stress tolerance via the activation of antioxidative pathways [48]. The accumulation of compatible osmolytes such as proline, proteins, and soluble sugars was enhanced by AMF inoculation under stress conditions (Figure 7). However, proline acts as an osmoprotectant that maintains the cellular water balance, thereby minimizing the negative effects of heat and drought on plant water status, under stress conditions. These osmolytes support plant tolerance to osmotic stress by stabilizing enzymes, membranes, and other cellular structures, even at high concentrations, without causing damage [53,54].

Previous studies have similarly shown that AMF can regulate osmolyte synthesis by modulating the metabolic pathways involved in proline production [55]. Elevated soluble sugar levels in mycorrhizal plants also contribute to osmotic adjustment and serve as an energy source supporting stress recovery [56]. Taken together, the results indicate that AMF act as natural bioprotectants that enhance both the physiological and biochemical resilience of lilies to drought and heat stress. By improving water status, boosting antioxidant defenses, and promoting osmotic adjustment, AMF effectively minimize metabolic disruptions caused by extreme conditions. Importantly, AMF application may also be cost-effective in floriculture because inoculation is typically applied once at planting and transplanting, and the resulting improvements in nutrient and water use efficiency can lower reliance on chemical fertilizers and other stress mitigation inputs while enhancing ornamental quality and market value. These findings highlight the potential of AMF as sustainable biofertilizers in floriculture, especially in regions facing increasing temperature variability and water scarcity due to climate change. Incorporating AMF inoculation into lily production systems could therefore contribute to climate-resilient ornamental horticulture and reduced dependence on chemical inputs.

## 5. Conclusions

This study demonstrates the promising role of AMF in improving the resilience of *Lilium* against drought and heat stress, with outcomes strongly influenced by host genotype and AMF inoculum. In the screening experiment, AMF strains viz, namely FM, RI, RIG, CE, DV, and MIX, showed distinct colonization and trait response patterns. Across multiple traits, DV and MIX provided the most consistent benefits in growth and colonization, whereas CE generally exhibited weaker colonization and smaller trait responses, with FM and RI often producing intermediate effects. Based on screening, Taiwan lily and MIX were advanced to the stress experiment to enable clearer treatment differentiation under controlled conditions. Under stress, combined drought + heat produced the strongest reductions in plant performance. AMF inoculation improved plant growth, while simultaneously reducing oxidative damage through enhanced antioxidant activity and osmotic regulation. Improved chlorophyll content and relative water status further highlight the contribution of AMF to maintaining physiological stability under stress conditions. Importantly, AMF and stress interactions were generally non-significant, indicating that AMF effects were mainly additive rather than stress dependent. Collectively, these findings show that AMF can serve as an eco-friendly biostimulant to support climate-resilient lily production.

## Figures and Tables

**Table 1:** Effects of different arbuscular mycorrhizal fungal (AMF) strains on floral traits of two *Lilium* species.

Plant Species	AMF	No. of Flower Buds	Flower Bud Length (mm)	Flower Bud Width (mm)	Flower Diameter (cm)
Taiwan lily	CK	1.67 ± 0.18 e	69.95 ± 7.54	11.52 ± 0.79 g	7.64 ± 0.62 f
	FM	2.37 ± 0.22 c–e	73.67 ± 5.39 ab	12.41 ± 0.68 fg	9.00 ± 0.69 d–f
	RI	2.17 ± 0.17 de	79.09 ± 3.79 a	13.49 ± 0.23 e–g	9.34 ± 0.52 c–f
	RIG	2.33 ± 0.17 de	76.50 ± 8.32 ab	14.64 ± 1.44 d–g	9.46 ± 0.76 c–f
	CE	2.11 ± 0.20 de	73.77 ± 4.77 ab	12.89 ± 0.91 fg	8.49 ± 0.98 ef
	DV	3.30 ± 0.30 b–d	81.61 ± 3.25 a	16.39 ± 1.13 b–g	10.12 ± 0.87 b–f
	MIX	3.87 ± 0.47 a–c	85.63 ± 6.99 a	15.45 ± 1.53 c–g	10.84 ± 0.60 b–f
Sorbonne	CK	3.33 ± 0.33 b–d	59.00 ± 5.20 b	15.67 ± 1.77 c–g	11.42 ± 1.17 a–f
	FM	4.67 ± 0.33 ab	75.00 ± 2.89 ab	21.00 ± 1.53 a–c	14.03 ± 0.58 a–c
	RI	4.03 ± 0.09 ab	72.00 ± 1.16 ab	19.67 ± 2.19 a–d	13.66 ± 1.24 a–d
	RIG	5.20 ± 0.42 a	77.33 ± 4.06 ab	22.33 ± 0.88 ab	14.74 ± 0.46 ab
	CE	4.17 ± 0.44 ab	67.00 ± 3.79 ab	17.67 ± 1.45 b–f	12.63 ± 0.96 a–e
	DV	5.33 ± 0.33 a	71.33 ± 5.79 ab	19.33 ± 1.45 a–e	13.77 ± 1.55 a–d
	MIX	5.13 ± 0.47 a	78.67 ± 3.53 ab	24.00 ± 2.08 a	15.66 ± 1.52 a

Note: Values are presented as mean ± standard error (n = 3). Different lowercase letters within a column denote statistically significant differences at *p* < 0.05 according to Tukey’s HSD test.

**Table 2:** Effects of different mycorrhizal strains on mycorrhizal colonization (%) in two *Lilium* species, the Taiwan lily and *Lilium* cv. Sorbonne.

Plant Species	AMF	Arbuscule (%)	Vesicle (%)	Total Colonization (%)
Taiwan lily	FM	43.33 ± 2.52 bc	59.50 ± 4.43 b–e	80.62 ± 2.87 ab
	RI	40.51 ± 2.74 cd	65.38 ± 2.91 bc	69.66 ± 3.28 a–c
	RIG	26.94 ± 3.25 fg	56.10 ± 2.89 c–f	63.96 ± 4.03 b–d
	CE	29.28 ± 1.11 e–g	47.28 ± 4.06 f	51.62 ± 5.59 cd
	DV	50.57 ± 2.24 b	68.32 ± 4.98 b	75.76 ± 4.67 ab
	MIX	73.27 ± 5.32 a	82.73 ± 5.54 a	89.83 ± 3.69 a
Sorbonne	FM	37.66 ± 4.57 c–e	52.50 ± 2.27 ef	64.33 ± 1.45 b–d
	RI	32.78 ± 2.51 d–f	58.34 ± 3.44 b–e	61.22 ± 5.54 b–d
	RIG	23.11 ± 2.08 fg	56.20 ± 2.49 c–f	59.63 ± 6.11 b–d
	CE	20.51 ± 1.69 g	36.41 ± 1.91 g	42.00 ± 4.59 d
	DV	41.86 ± 4.26 b–d	54.50 ± 3.42 d–f	61.33 ± 2.41 b–d
	MIX	39.26 ± 1.96 c–e	63.42 ± 4.68 b–d	79.29 ± 4.68 ab

Note: Values are presented as mean ± standard error (n = 3). Different lowercase letters within a column denote statistically significant differences at *p* < 0.05 according to Tukey’s HSD test.

## Data Availability

All data supporting the findings of this study are included within the article and its Supplementary Materials. Additional datasets or raw data are available from the corresponding author upon reasonable request.

## Supplementary Materials

The following supporting information can be downloaded at: https://www.mdpi.com/article/10.3390/plants15050767/s1, Figure S1: Visual comparison of bulb and root morphology in two lily cultivars (Taiwan and Sorbonne) under different arbuscular mycorrhizal fungal (AMF) treatments. Figure S2: Representative images of root morphology and bulb development of Taiwan lily under control (Ck; no stress), drought (D), heat (H), and combined drought + heat (HD) stress with AMF (M+) and without AMF (M-) inoculation. Table S1: Effects of different arbuscular mycorrhizal fungal (AMF) strains on growth parameters of two *Lilium* species, the Taiwan lily and *Lilium* cv. Sorbonne. Table S2: Effects of different arbuscular mycorrhizal fungal (AMF) strains on root and bulb traits of two *Lilium* species, Taiwan lily and *Lilium* cv. Sorbonne. Table S3: Summary of analysis of variance (ANOVA) for the effects of arbuscular mycorrhizal fungi (AMF) inoculation, lily species, and their interaction on lily growth. Table S4: List of chemicals and reagents used in this study.
