# Supplementary material for: Harnessing Arbuscular Mycorrhizal Symbiosis to Enhance Growth and Resilience to Combined Drought and Heat Stress in Lily (Lilium spp.)
Source: Plants (Basel). 2026 Mar 2;15(5):767. doi: 10.3390/plants15050767 (PMC12987297; doi:10.3390/plants15050767)
Supplement: Supplementary file 1 [file plants-15-00767-s001.zip › plants-4035235-supplementary.pdf]

Supplementary material

Harnessing Arbuscular Mycorrhizal Symbiosis to Enhance Growth and Resilience to Combined Drought and Heat Stress in Lily (Lilium spp.)

Hafiz Athar Hussain[a], Zhanhuai Liang[a], Shujaat Hussain[b], Jianghui Luo[a], Shunzhao Sui[a]*, Daofeng Liu[a]*

[a] *Chongqing Engineering Research Center for Floriculture, Key Laboratory of Agricultural Biosafety and Green Production of Upper Yangtze River (Ministry of Education), College of Horticulture and Landscape Architecture, Southwest University, Chongqing 400715, China.*
[b] *Institute of Horticultural Sciences, University of Agriculture, Faisalabad 38040, Punjab, Pakistan.*

*Corresponding: szcq@swu.edu.cn; liu19830222@163.com

Supporting figures and tables (Figures S1-S2; Tables S1-S4)

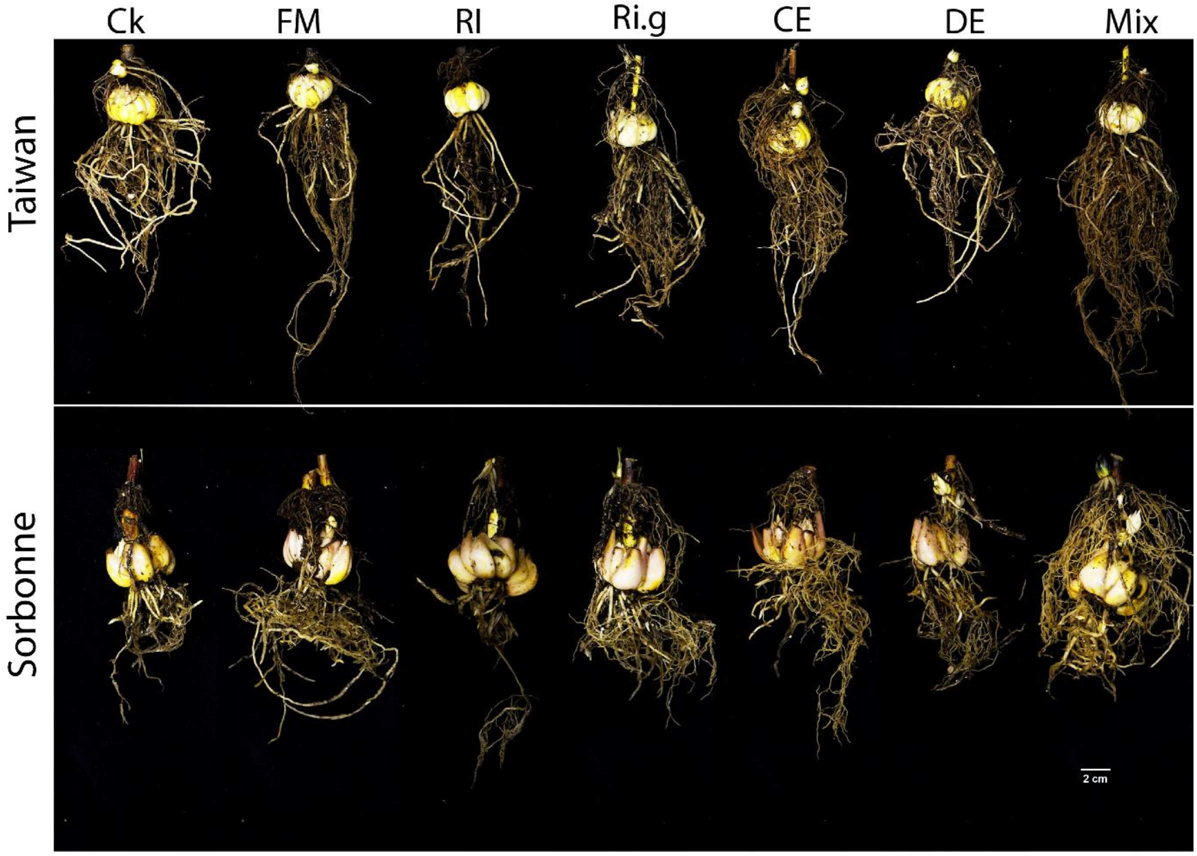

Figure S1: Visual comparison of bulb and root morphology in two lily cultivars (Taiwan and Sorbonne) under different arbuscular mycorrhizal fungal (AMF) treatments.

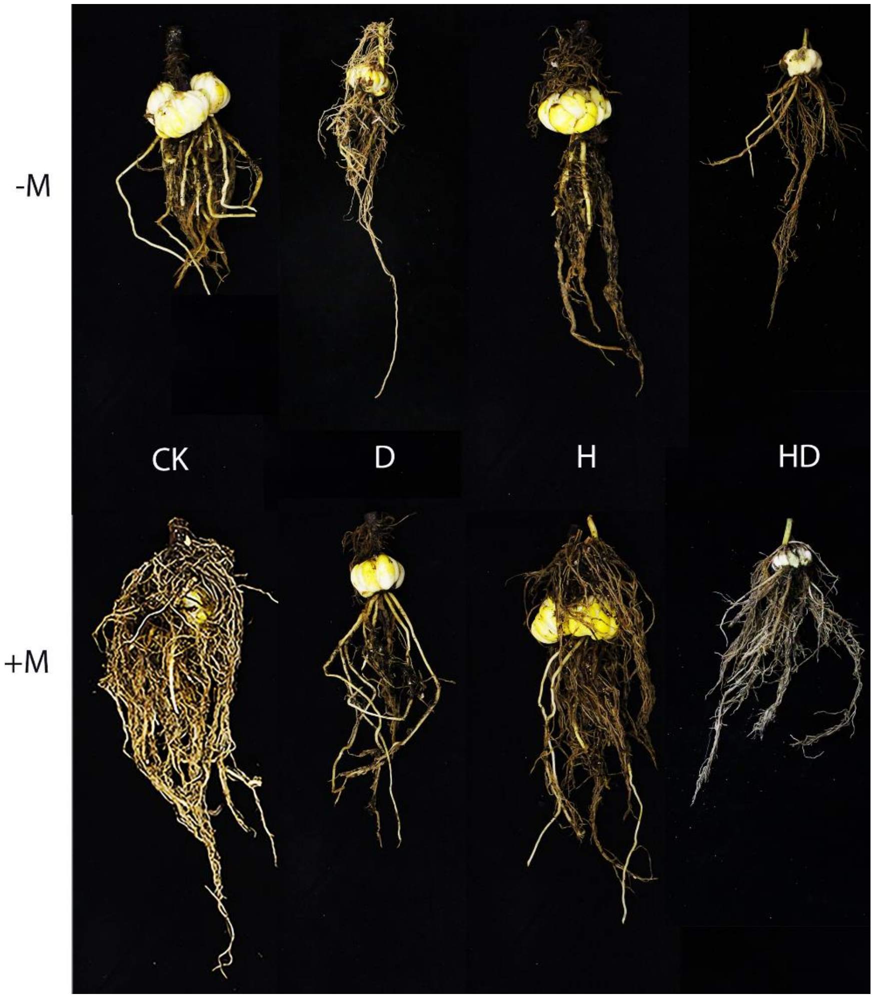

Figure S2: Representative images of root morphology and bulb development of Taiwan lily under drought (D), heat (H), and combined drought + heat (HD) stress with AMF (M+) and without AMF (M-) inoculation.

Table S1: Effects of different arbuscular mycorrhizal fungal (AMF) strains on growth parameters of two Lilium species, the Taiwan lily and Lilium cv. Sorbonne.

Plant species	AMF	Plant height (cm)	Stem diameter (mm)	Shoot dry weight (g)	No. of leaves
Taiwan	CK	53.89±3.96d	21.72±0.39a	3.05±0.28e	47.17±3.12b
	FM	61.44±2.28cd	22.31±0.35a	3.90±0.30b-e	64.17±4.35ab
	RI	58.05±1.79cd	21.91±0.19a	3.62±0.39e	59.17±6.17ab
	RIG	57.33±1.77cd	22.43±0.34a	3.27±0.37e	61.83±4.59ab
	CE	60.89±3.35cd	21.88±0.46a	3.42±0.32e	60.67±2.62ab
	DV	62.83±2.05cd	23.01±0.14a	3.28±0.31e	67.33±6.02a
	MIX	64.00±2.37bcd	23.16±0.25a	3.68±0.27de	76.63±3.28a
Sorbonne	CK	72.17±7.98a-d	20.41±2.08a	3.78±0.24cde	17.83±1.30c
	FM	76.83±7.44abc	23.75±1.65a	4.40±0.40a-e	21.83±1.48c
	RI	73.83±4.87a-d	23.05±0.21a	3.99±0.38b-e	22.00±1.16c
	RIG	85.00±5.30ab	22.63±0.32a	5.93±0.54a	19.83±1.48c
	CE	72.56±2.38a-d	23.01±0.34a	5.48±0.11a-d	20.83±2.24c
	DV	92.33±4.49a	24.18±0.27a	5.69±0.36ab	18.83±0.44c
	MIX	76.17±1.74abc	23.32±0.81a	5.54±0.45abc	22.17±1.59c

Note: Values are presented as mean ± standard error (n = 3). Different lowercase letters within a column denote statistically significant differences at $p < 0.05$ according to Tukey's HSD test.

Table S2: Effects of different arbuscular mycorrhizal fungal (AMF) strains on root and bulb traits of two Lilium species, Taiwan lily and Lilium cv. Sorbonne.

Plant species	AMF	Root length (cm)	Root dry weight (g)	Perimeter of bulb (cm)	No of bulblets
Taiwan lily	CK	17.17±3.25b-e	6.00±0.22d	10.84±1.09c	0.83±0.09g
	FM	25.25±2.75a	6.26±0.57cd	12.86±1.05bc	2.67±0.33de
	RI	17.78±3.60b-e	7.49±0.26bcd	12.86±1.05bc	1.33±0.17fg
	RIG	23.67±3.82 ab	7.62±0.20bcd	12.38±0.66bc	2.33±0.17def
	CE	20.00±2.29a-d	6.85±0.25bcd	12.71±0.05bc	1.33±0.17fg
	DV	21.33±1.53abc	7.85±0.90bcd	12.71±0.05bc	2.67±0.17de
	MIX	24.08±2.24ab	7.72±0.80bcd	14.80±1.25ab	3.00±0.29cde
Sorbonne	CK	8.83±0.84f	8.47±0.97a-d	17.23±0.46a	1.83±0.17efg
	FM	12.83±0.60def	12.07±1.10a	17.65±0.16a	4.17±0.44abc
	RI	13.52±1.55def	9.50±1.04a-d	18.22±0.94a	2.47±0.26def
	RIG	12.43±0.54ef	10.27±0.94abc	17.50±0.48a	3.50±0.29bcd
	CE	16.33±1.64cde	12.13±1.04a	18.38±0.85a	3.22±0.22bcd
	DV	14.27±1.40c-f	11.00±0.99ab	17.93±0.12a	5.11±0.11a
	MIX	17.00±2.08b-e	10.37±0.86abc	17.60±0.38a	4.33±0.33ab

Note: Values are presented as mean ± standard error (n = 3). Different lowercase letters within a column denote statistically significant differences at $p < 0.05$ according to Tukey’s HSD test.

Table S3: Summary of analysis of variance (ANOVA) for the effects of arbuscular mycorrhizal fungi (AMF) inoculation, lily species, and their interaction on lily growth

Traits	AMF	Lily Species	AMF× Lily Species
Plant height	2.46ns	71.89***	1.62ns
Shoot diameter	2.15ns	1.80ns	0.76ns
Shoot dry weight	4.41**	64.45***	2.59*
No of leaves	4.20**	523.11***	2.78*
Root length	6.22***	107.88***	2.75*
Root dry weight	1.86ns	63.25***	1.71ns
Perimeter of bulb	1.63ns	165.12***	1.15ns
No of bulblets	32.65***	137.87***	2.30ns
No of flower buds	12.10***	170.06***	1.52ns
Flower bud length	4.19**	7.6 6*	0.82ns
Flower bud width	6.31***	99.07***	1.90ns
Flower diameter	3.32*	81.71***	0.23ns
Arbuscule	104.51***	79.52***	16.69***
Vesicle	1.60ns	54.81***	20.20***
Total colonization	17.16***	15.81***	0.47ns

F values are present with: ns, not significant, *P<0.05, **P<0.01., ***P<0.001. AMF: arbuscular mycorrhizal fungi

Table S4: List of chemicals and reagents used in this study

Reagent	Application	Manufacturer	Purity
Trypan Blue	Root staining for mycorrhizal colonization	Coolaber Science & Technology Co., Ltd, Beijing, China	≥40% dye content
Lactic acid	Component of lactoglycerol mounting/staining solution	Chengdu Kelong Chemical Co., Ltd, Chengdu, China	85-90%
Glycerol	Component of lactoglycerol mounting/staining solution	Chengdu Kelong Chemical Co., Ltd, Chengdu, China	99.%
Acetic acid	For bleaching solution in H_2O_2	Chengdu Kelong Chemical Co., Ltd, Chengdu, China	≥99%
Ethanol	For bleaching solution in DAB staining and Chl	Chengdu Kelong Chemical Co., Ltd, Chengdu, China	≥99%
Acetone	H_2O_2 assay and Chl	Chengdu Kelong Chemical Co., Ltd, Chengdu, China	≥99%
2,3,5-Triphenyltetrazolium chloride (TTC)	Root activity assay (TTC reduction)	Coolaber Science & Technology Co., Ltd, Beijing, China	≥98%
Ethyl acetate	Root activity assay	Chengdu Kelong Chemical Co., Ltd, Chengdu, China	≥99%
Trichloroacetic acid (TCA)	MDA extraction and assay reagent	Chengdu Kelong Chemical Co., Ltd, Chengdu, China	≥99%
Thiobarbituric acid (TBA)	MDA (TBARS) assay reagent	Aladdin Industrial Corporation, Shanghai, China	≥98%
Nitroblue tetrazolium chloride (NBT)	Histochemical staining and SOD activity assay	Nanjing Duly Biotech Co., Ltd, Nanjing, China	≥ 98
Hydroxylamine hydrochloride	Superoxide quantification (hydroxylamine method)	Coolaber Science & Technology Co., Ltd, Beijing, China	>96%
Sulfanilic acid	Superoxide quantification (diazotization step)	Aladdin Industrial Corporation, Shanghai, China	≥99%
alpha-Naphthylamine	Superoxide quantification (coupling reagent)	Shanghai Macklin Bio-chemical Co., Ltd, Shanghai, China	99.%
3,3′-Diaminobenzidine (DAB)	Histochemical staining of H_2O_2	Coolaber Science & Technology Co., Ltd, Beijing, China	≥98%
Ethylenediaminetetraacetic acid (EDTA)	SOD extraction/reaction buffer component	Chengdu Kelong Chemical Co., Ltd, Chengdu, China	≥99%
L-Methionine	SOD activity assay component	Coolaber Science & Technology Co., Ltd, Beijing, China	≥98.5%
Riboflavin	SOD activity assay (photochemical system)	Shanghai Macklin Biochemical Co., Ltd, Shanghai, China	98%
Sulfosalicylic acid	Proline extraction	Shanghai Macklin Bio-chemical Co., Ltd, Shanghai, China	≥98%
Ninhydrin	Proline color development (acid ninhydrin method)	Chengdu Kelong Chemical Co., Ltd, Chengdu, China	95%
L-Proline	Standard for proline quantification	Shanghai Macklin Biochemical Co., Ltd, Shanghai, China	99%
